# Crystal structure and Hirshfeld surface analyses, crystal voids, inter­action energy calculations and energy frameworks of (*E*)-2-[(pyren-1-yl­methylidene)amino]­ethanol

**DOI:** 10.1107/S2056989025004451

**Published:** 2025-06-10

**Authors:** Naser E. Eltayeb, Tuncer Hökelek, Jamal Lasri

**Affiliations:** a Department of Chemistry Rabigh College of Science and Arts King Abdulaziz University,Jeddah 21589 Saudi Arabia; b Department of Chemistry, Faculty of Pure and Applied Sciences, International University of Africa, Khartoum 2469, Sudan; c Department of Physics, Hacettepe University, 06800 Beytepe, Ankara, Türkiye; Universidade Federal do ABC, Brazil

**Keywords:** pyrene Schiff base, Hirshfeld surface, energy framework analysis, hydrogen bond, π-stacking, crystal structure

## Abstract

The title compound, C_19_H_15_NO, contains a pyrene ring system, consisting of four fused benzene rings arranged in a planar configuration. In the crystal, inter­molecular O—H⋯N hydrogen bonds link the mol­ecules into infinite chains along the *c*-axis direction. π–π stacking inter­actions between the benzene rings of adjacent mol­ecules help to consolidate the three-dimensional architecture. A Hirshfeld surface analysis indicates that the most important contributions for the crystal packing are from H⋯H (56.4%), H⋯C/C⋯H (16.6%) and C⋯C (15.8%) inter­actions. The volume of the crystal voids and the percentage of free space were calculated to be 76.07 Å^3^ and 5.79%, showing that there is no large cavity in the crystal packing. An evaluation of the electrostatic, dispersion and total energy frameworks indicates that the dispersion energy contribution is dominant while hydrogen bonding, π⋯π and van der Waals inter­actions are the dominant inter­actions in the crystal packing.

## Abstract 2

The title compound contains a pyrene ring system consisting of four fused benzene rings arranged in a planar configuration. In the crystal, inter­molecular O—H⋯N hydrogen bonds link the mol­ecules into infinite chains along the *c*- axis direction. π–π stacking inter­actions between the benzene rings of adjacent mol­ecules help to consolidate the three-dimensional architecture.

## 1. Chemical context

Schiff bases have garnered significant attention in coordination and medicinal chemistry and in materials science due to their structural versatilities, ease of syntheses, and diverse applications (Gupta & Sutar, 2008 ▸). The abilities of Schiff bases to act as chelating ligands by coordinating various transition-metal ions make them valuable in catalysis, bio­organic chemistry, and in the development of metallodrugs (da Silva *et al.*, 2011 ▸). Additionally, their biological activities including anti­microbial (Malik *et al.*, 2018 ▸) and anti­oxidant properties (Kumar *et al.*, 2017 ▸) have made them promising candidates for pharmaceutical applications. Schiff bases also find utility in industrial processes such as corrosion inhibition (Omar *et al.*, 1986 ▸), polymer stabilization (Sabaa *et al.*, 2009 ▸), and as sensors for metal ion detection (Alam *et al.*, 2023 ▸). Currently, our research program focuses on the syntheses and evaluations of anti­cancer activities of Schiff base type compounds (Lasri *et al.*, 2018 ▸, 2023*a* ▸,*b* ▸, 2024 ▸; Eltayeb *et al.*, 2020*a* ▸,*b* ▸). Herein, we report the synthesis, mol­ecular and crystal structures, Hirshfeld surface analysis, crystal voids, inter­action energies and energy frameworks of the title compound (I).

## 2. Structural commentary

**Figure 1:**
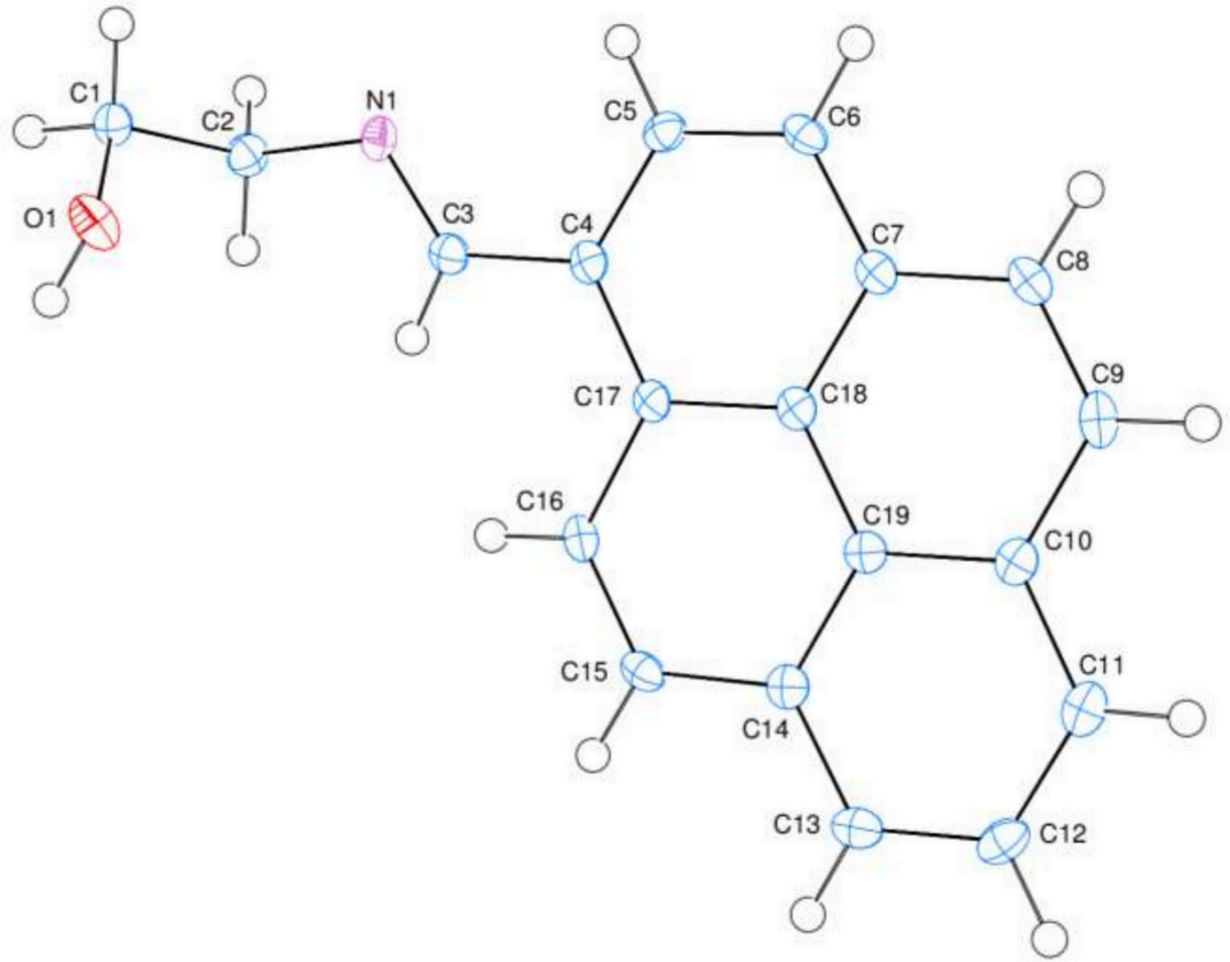
The title mol­ecule with atom-numbering scheme and 50% probability ellipsoids.

The title compound contains a planar pyrene ring system, consisting of four fused benzene rings [*A* (C4–C17/C18), *B* (C7–C10/C18/C19), *C* (C10–C14/C19) and *D* (C14–C19)] arranged in a planar configuration (Fig. 1 ▸), where atom C3 is 0.0255 (13) Å away from the best least-squares plane of the ring system. The C2—N1—C3—C4, N1—C3—C4—C5, N1—C3—C4—C17 and O1—C1—C2—N1 torsion angles are −178.71 (11), −9.67 (19), 171.23 (12) and −61.54 (14)°, respectively. There are no unusual bond distances or inter-bond angles in the mol­ecule.
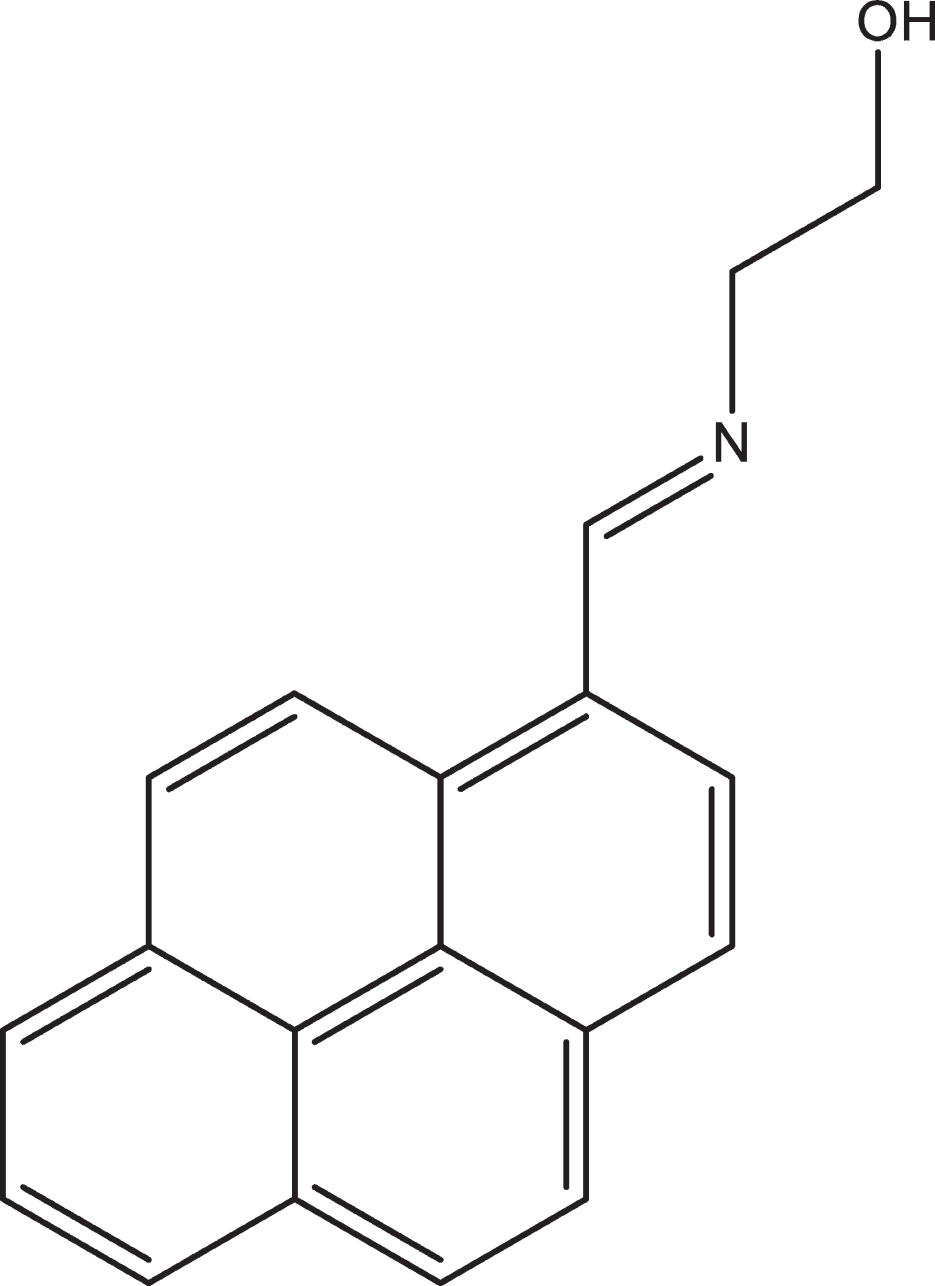


## 3. Supra­molecular features

**Figure 2:**
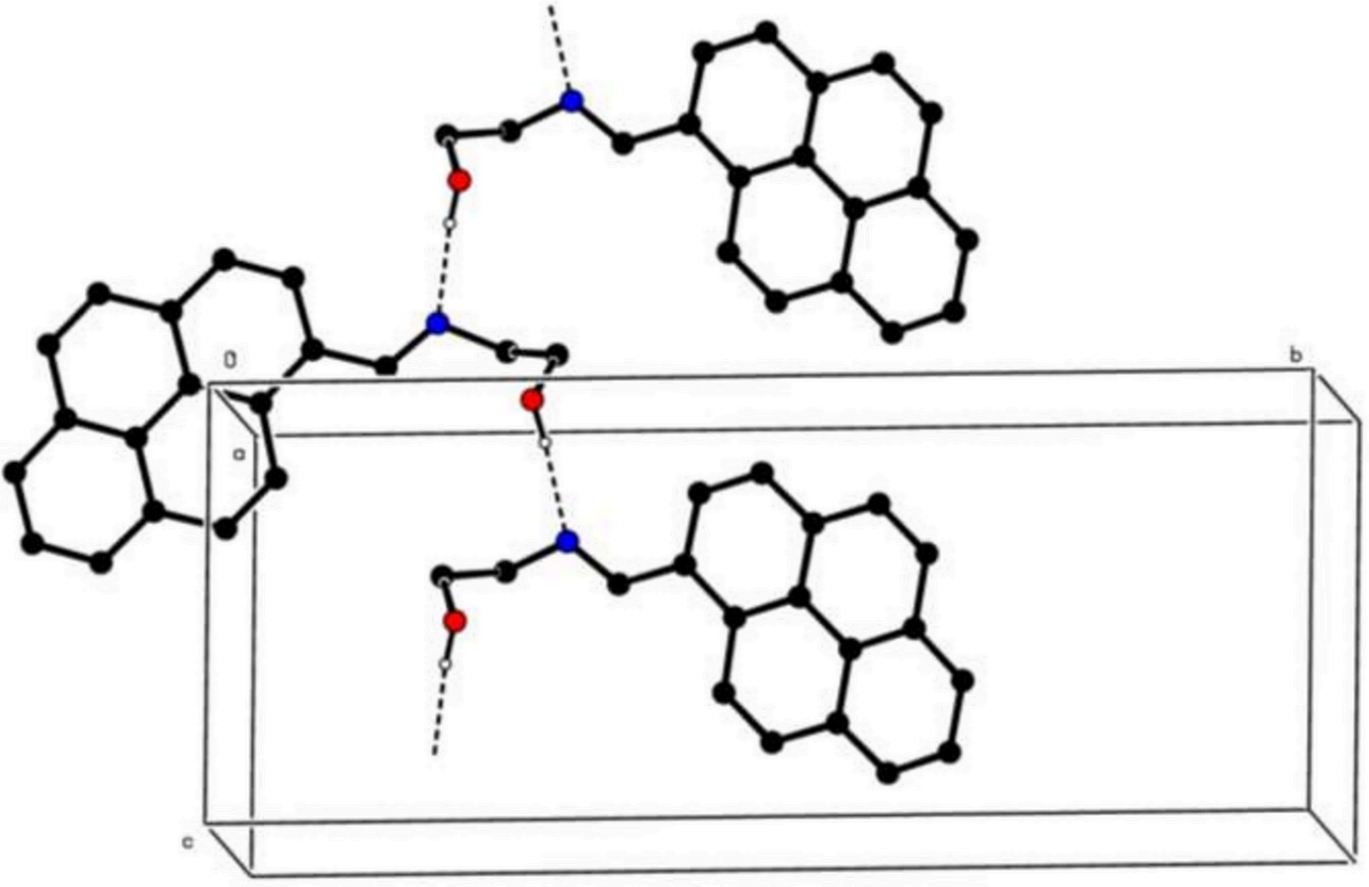
A partial packing diagram viewed down the *a*-axis direction. Inter­molecular O—H⋯N hydrogen bonds are shown as dashed lines. H atoms not involved in these inter­actions have been omitted for clarity.

In the crystal, inter­molecular O—H⋯N hydrogen bonds (Table 1 ▸) link the mol­ecules into infinite chains along the *c*-axis direction (Fig. 2 ▸). π–π stacking inter­actions occur between the benzene rings of adjacent mol­ecules with the inter-centroid distances of 4.3657 (18) Å [between *A* rings, α = 0.02 (6)° and slippage = 2.632], 3.6343 (16) Å [between *A* and *B* rings, α = 0.05 (6)° and slippage = 1.079], 3.7953 (16) Å [between *A* and *C* rings, α = 1.90 (6)° and slippage = 1.663], 3.6538 (16) Å [between *A* and *D* rings, α = 1.10 (6)° and slippage = 1.418], 4.4161 (18) Å [between *B* rings, α = 0.00 (6)° and slippage = 2.727], 3.8279 (16) Å [between *B* and *D* rings, α = 0.14 (6)° and slippage = 1.609], 4.1054 (17) Å [between *D* rings, α = 0.00 (6)° and slippage = 2.367] and may help to consolidate the three-dimensional architecture. No C—H⋯π(ring) inter­actions are identified.

## 4. Hirshfeld surface analysis

**Figure 3:**
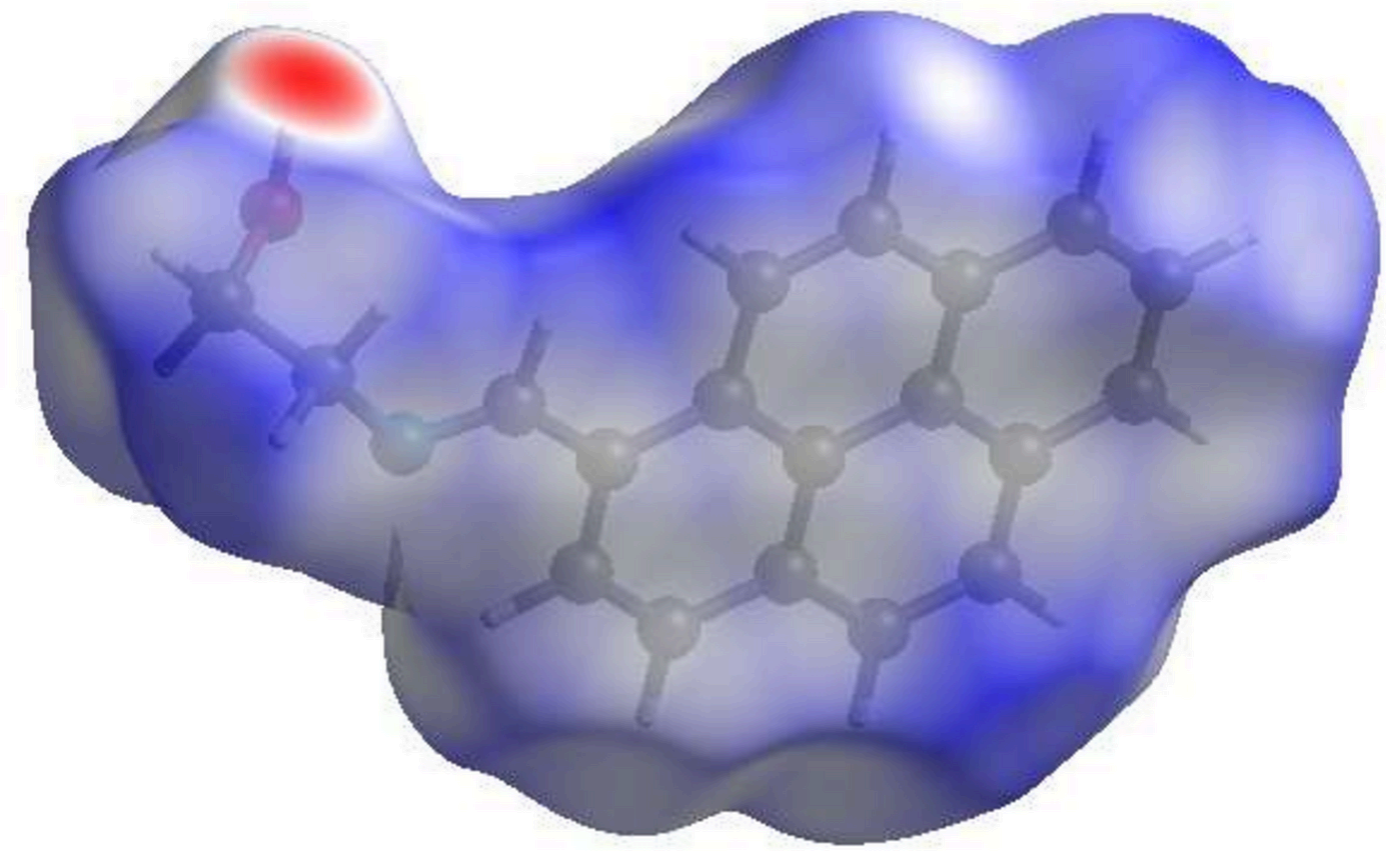
View of the three-dimensional Hirshfeld surface of the title compound plotted over *d*_norm_.

**Figure 4:**
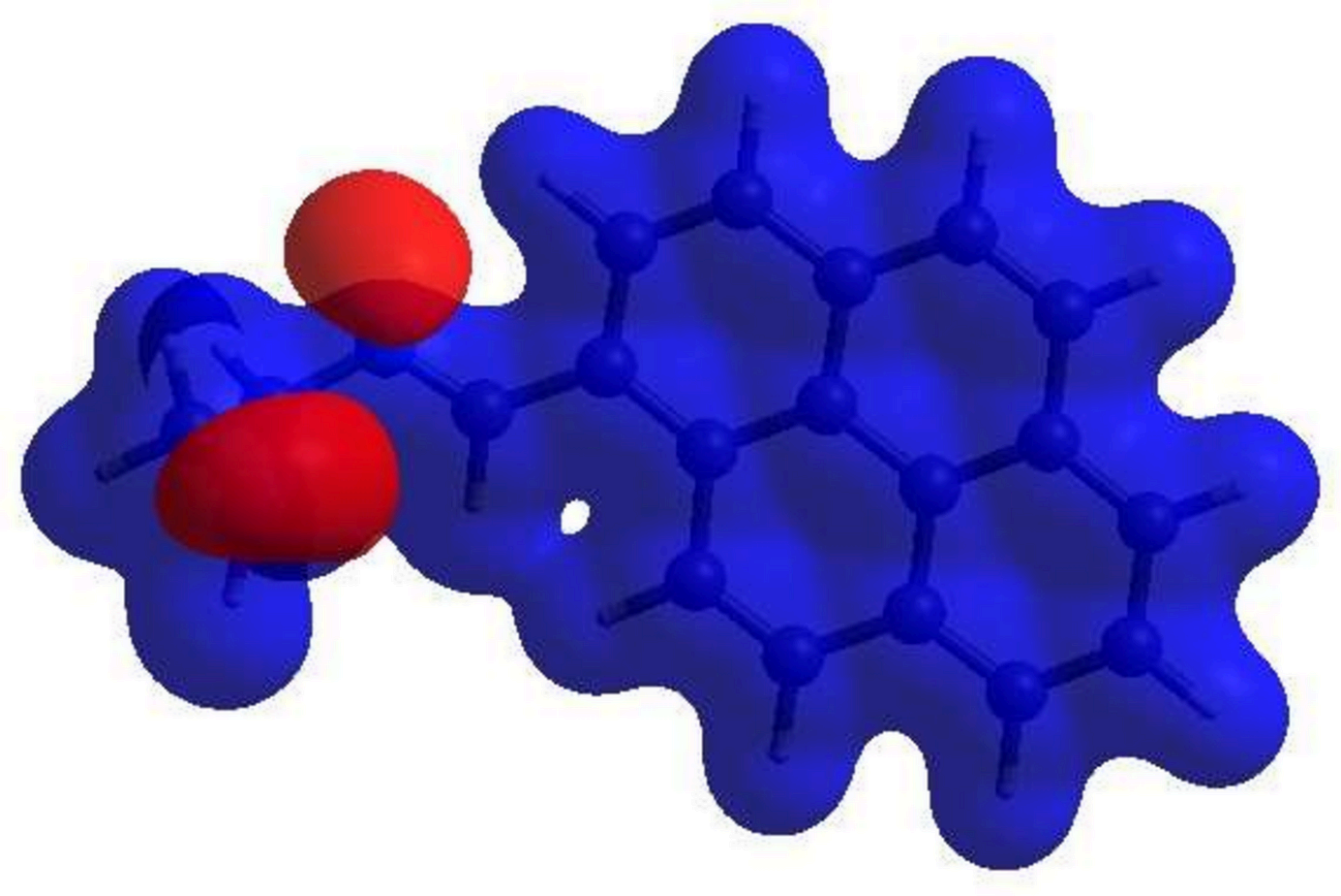
View of the Hirshfeld surface of the title compound plotted over electrostatic potential energy in the range of −0.0500 to 0.0500 a.u. using the STO-3 G basis set at the Hartree–Fock level of theory. Hydrogen-bond donors and acceptors are shown as blue and red regions around the atoms corresponding to positive and negative potentials, respectively.

**Figure 5:**
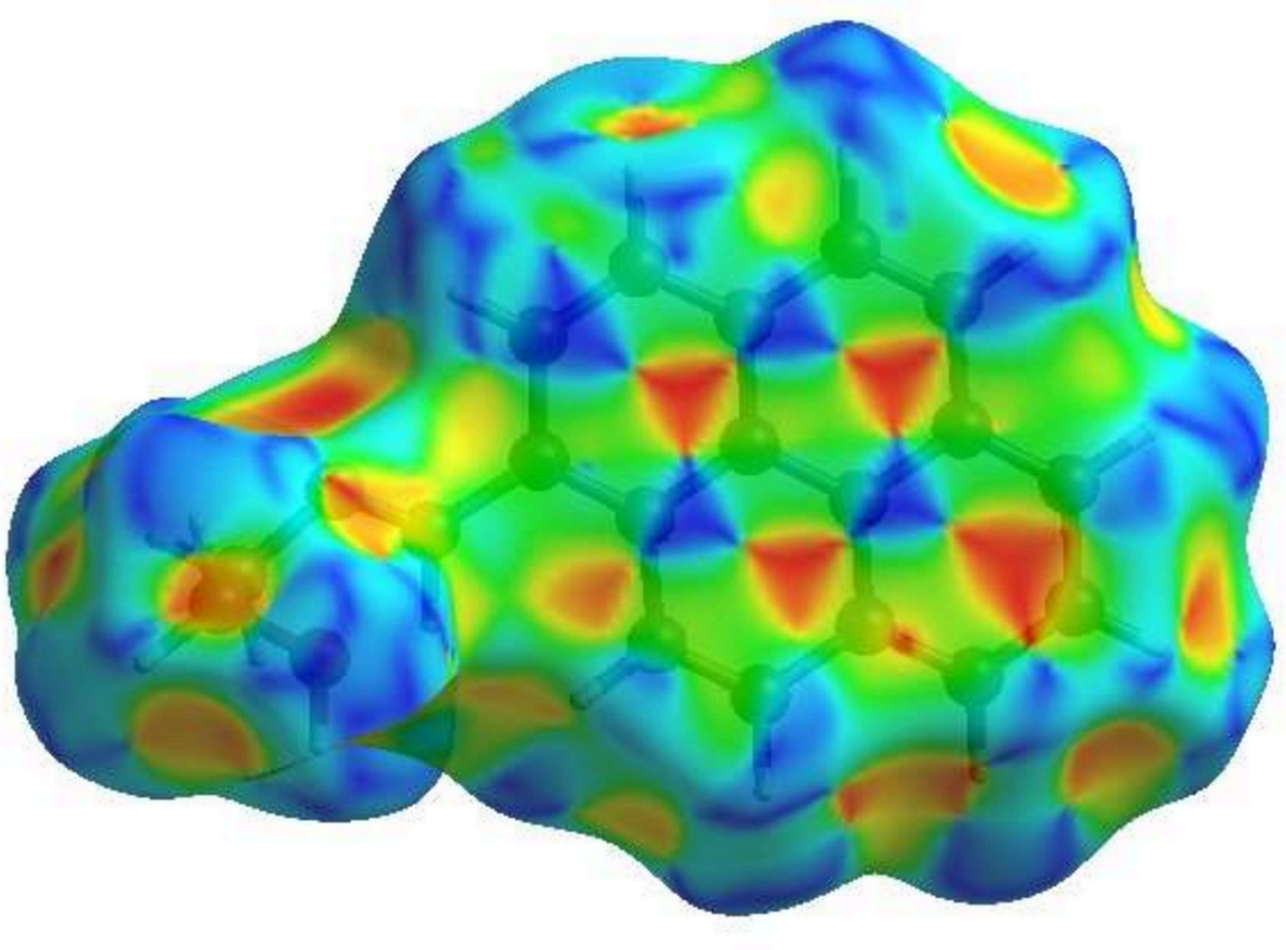
Hirshfeld surface of the title compound plotted over shape-index.

**Figure 6:**
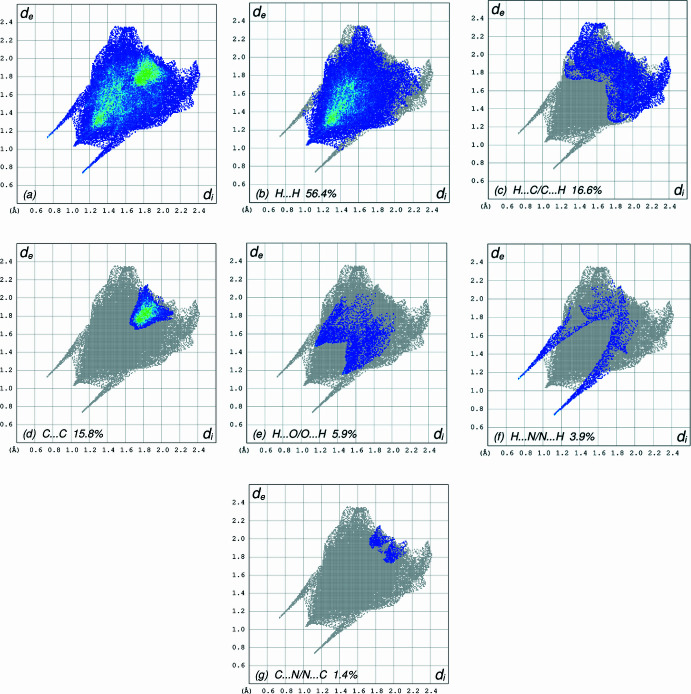
The full two-dimensional fingerprint plots for the title compound, showing (*a*) all inter­actions, and delineated into (*b*) H⋯H, (*c*) H⋯C/C⋯H, (*d*) C⋯C, (*e*) H⋯O/O⋯H, (*f*) H⋯N/N⋯H and (*g*) C⋯N/N⋯C, inter­actions. The *d*_i_ and *d*_e_ values are the closest inter­nal and external distances (in Å) from given points on the Hirshfeld surface contacts.

**Figure 7:**
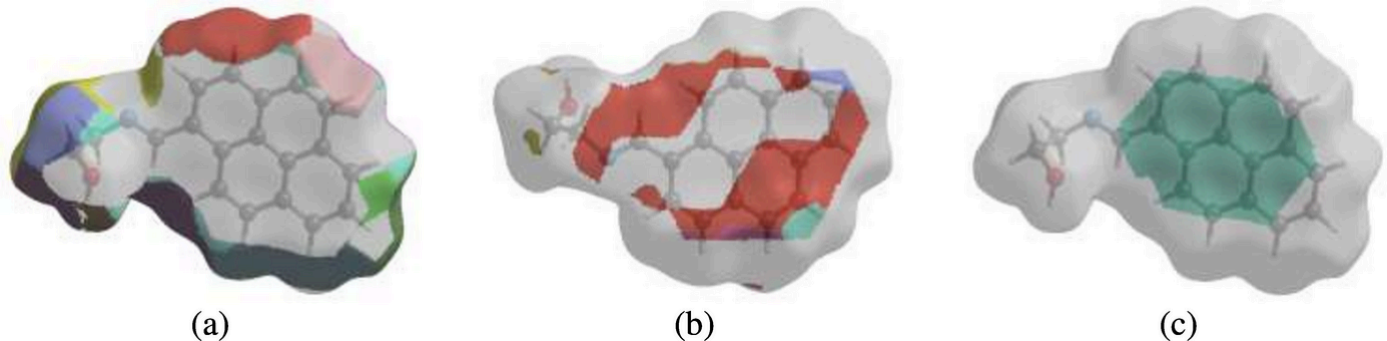
The Hirshfeld surface representations of contact patches plotted onto the surface for (*a*) H⋯H, (*b*) H⋯C/C⋯H and (*c*) C⋯C inter­actions.

A Hirshfeld surface (HS) analysis (Hirshfeld, 1977 ▸; Spackman & Jayatilaka, 2009 ▸) was carried out using *Crystal Explorer 17.5* (Spackman *et al.*, 2021 ▸) to clarify the inter­molecular inter­actions (Table 2 ▸) in the crystal of the title compound (I). The contact distances (Table 2 ▸) (Venkatesan *et al.*, 2016 ▸) are shown in Fig. 3 ▸, where the bright-red spots correspond to the respective donors and/or acceptors; they also appear as blue and red regions in Fig. 4 ▸ corresponding to positive and negative potentials (Spackman *et al.*, 2008 ▸; Jayatilaka *et al.*, 2005 ▸). Fig. 5 ▸ shows only the presence of the π–π inter­actions in (I). According to the 2D fingerprint plots (McKinnon *et al.*, 2007 ▸), the inter­molecular H⋯H (Table 2 ▸), H⋯C/C⋯H and C⋯C (Table 2 ▸) contacts make important contributions to the HS of 56.4%, 16.6% and 15.8%, respectively (Fig. 6 ▸). Their contact patches are also plotted onto the surface as shown in Fig. 7 ▸, suggesting that van der Waals inter­actions and hydrogen bonding play the major roles in the crystal packing (Hathwar *et al.*, 2015 ▸).

## 5. Crystal voids

**Figure 8:**
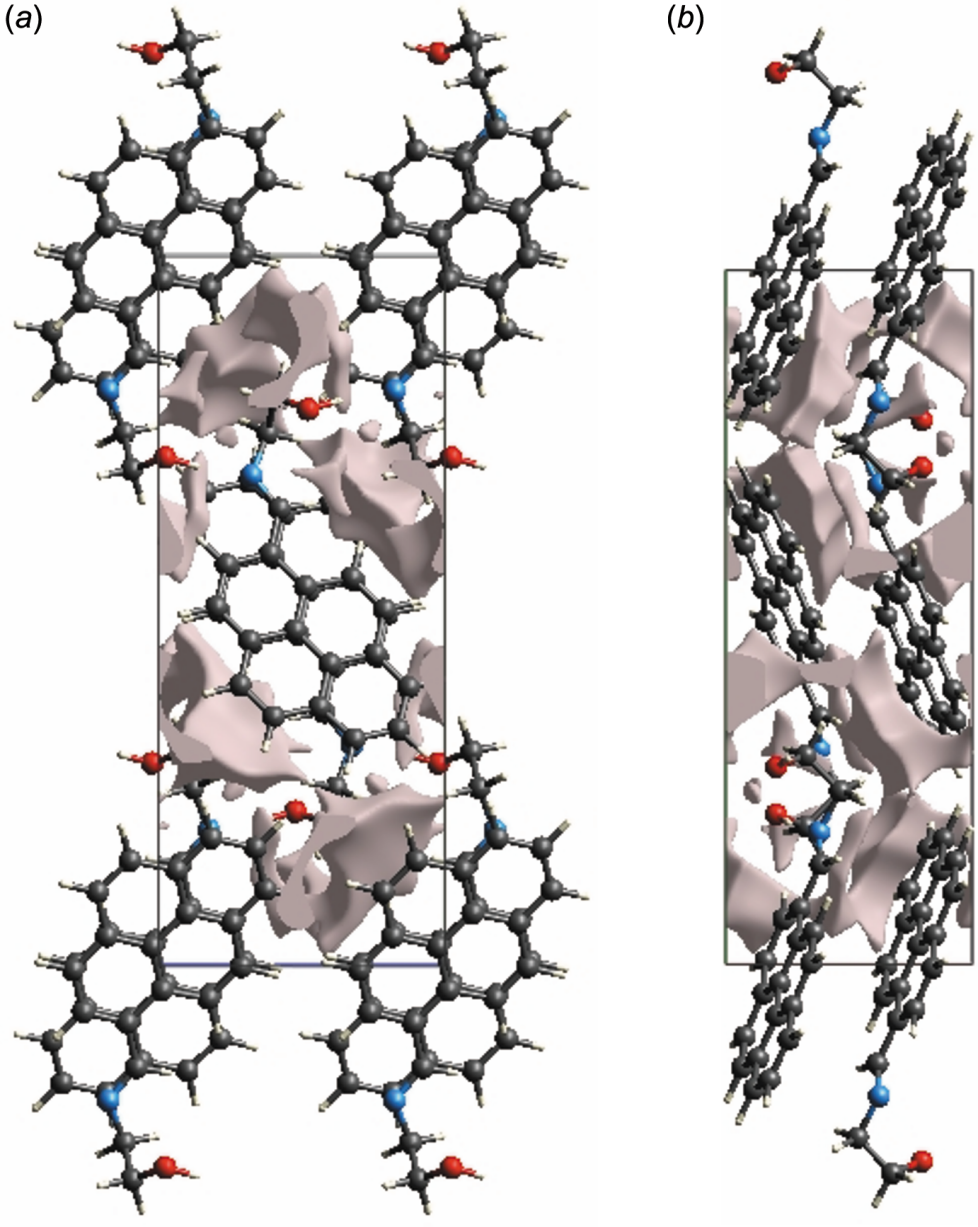
Graphical views of voids in the crystal packing of the title compound along the (*a*) *a*-axis and (*b*) *c*-axis directions.>

If the mol­ecules are tightly packed and an applied external mechanical force does not easily break the crystal, then the crystal packing does not result in significant voids. A void analysis was performed by adding up the electron densities of the spherically symmetric atoms contained in the asymmetric unit (Turner *et al.*, 2011 ▸). The volume of the crystal voids (Fig. 8 ▸) and the percentage of free space in the unit cell are calculated as 76.07 Å^3^ and 5.79%, respectively, indicating that the crystal packing is compact.

## 6. Inter­action energy calculations and energy frameworks

**Figure 9:**
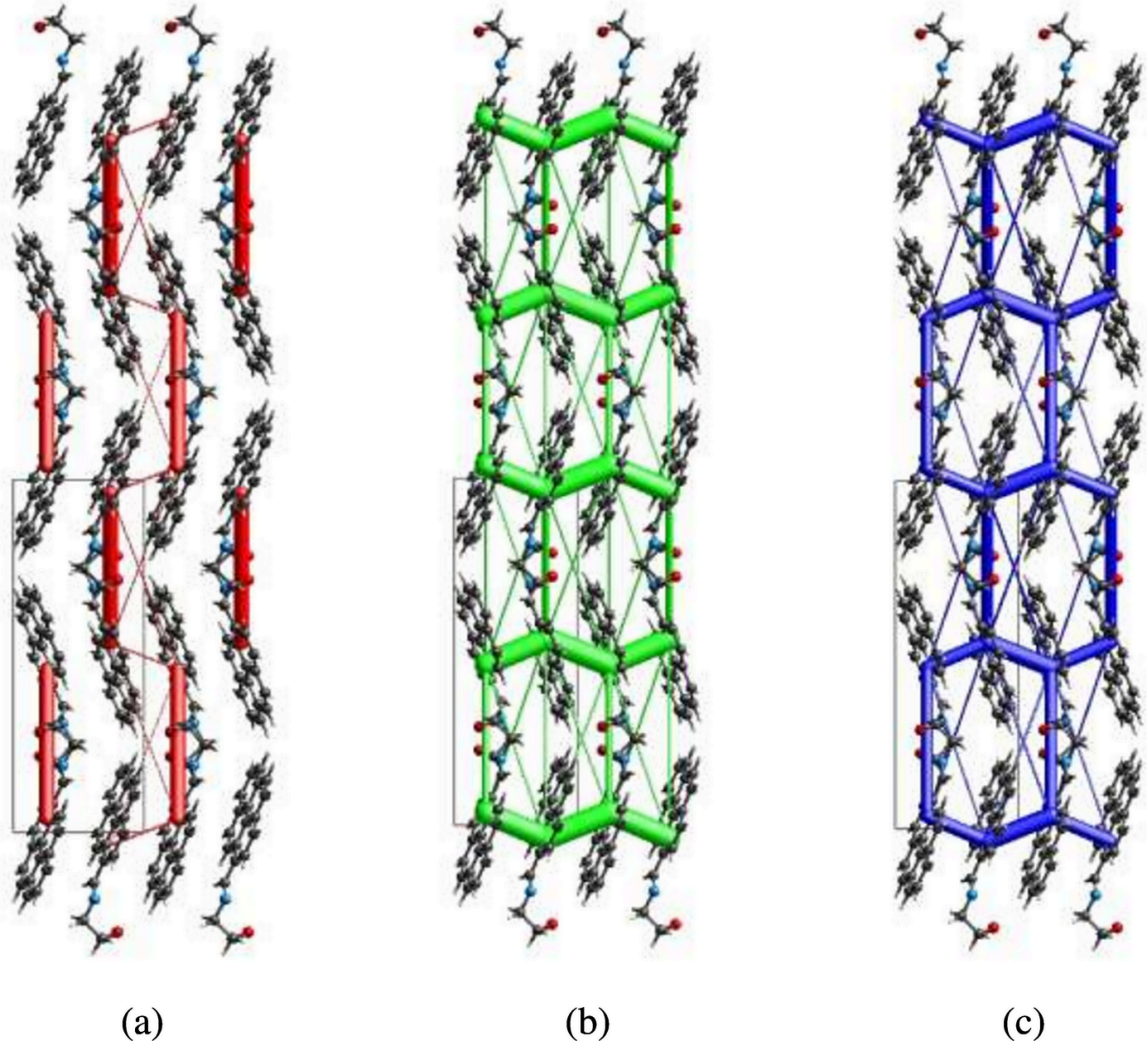
The energy frameworks for a cluster of mol­ecules of the title compound viewed down the *c*-axis showing the (*a*) electrostatic energy, (*b*) dispersion energy and (*c*) total energy diagrams. The cylindrical radius is proportional to the relative strength of the corresponding energies and they were adjusted to the same scale factor of 80 with cut-off value of 5 kJ mol^−1^ within 2 × 2 *X* 2 unit cells.

The CE–B3LYP/6–31G(d,p) energy model available in *Crystal Explorer 17.5* (Spackman *et al.*, 2021 ▸) was used to calculate the inter­molecular inter­action energies. Hydrogen-bonding inter­action energies (in kJ mol^−1^) were calculated to be −9.8 (*E*_ele_), −14.2 (*E*_pol_), −103.3 (*E*_dis_), 52.1 (*E*_rep_) and −70.0 (*E*_tot_) for O1—H1⋯N1. Energy frameworks combine the calculation of inter­molecular inter­action energies with a graphical representation of their magnitude (Turner *et al.*, 2015 ▸). Energy frameworks were constructed for *E*_ele_ (red cylinders), *E*_dis_ (green cylinders) and *E*_tot_ (blue cylinders) (Fig. 9 ▸*a*, *b* and *c*), and their evaluation indicates that the stabilization is dominated *via* the dispersion energy contributions in the crystal structure of (I).

## 7. Synthesis and crystallization

To a solution of 1-pyrenecarboxaldehyde (0.230 g, 1.0 mmol) in ethanol (25 ml) was added 2-amino­ethanol (0.073 g, 1.2 mmol) and the reaction mixture was refluxed for 4 h. The reaction mixture was cooled down to room temperature for precipitation, and then filtered. The precipitate was washed with cold ethanol and dried in air. Yellow crystals suitable for X-ray analysis were obtained by slow evaporation of an ethanol solution. Yield: 80%. FT–IR (cm^−1^): 3440 (OH), 1631 (C=N), 1594 (C=C). ^1^H NMR (CDCl_3_): 9.34 (*s*, 1H, N=CH), 9.12 (*d*, 1H, *J*_H–H_ = 9.6 Hz, pyren­yl), 8.56 (*d*, 1H, *J*_H–H_ = 7.8 Hz, pyren­yl), 8.36 (*m*, 7H, pyren­yl), 4.73 (*t*, *J*_H–H_ = 5.4 Hz, OH), 3.87 (*dd*, 4H, *J*_H–H_ = 4.8 and 5.4 Hz, CH_2_). ^13^C NMR (CDCl_3_): 161.83, 131.23, 128.75, 128.68, 127.41, 126.21, 125.97, 125.70, 124.92, 122.38, 64.20, 62.70; HRMS: *m*/*z*: 273.11 [M]^+^. Analysis calculated (%) for C_19_H_15_NO: C 83.49, H 5.53, N 5.12; found C 83.47, H 5.51, N 5.10.

## 8. Refinement

Crystal data, data collection and structure refinement details are summarized in Table 3 ▸. The OH hydrogen atom was located in a difference-Fourier map, and refined isotropically. The C-bound hydrogen-atom positions were calculated geometrically at distances of 0.93 Å (for aromatic CH) and 0.97 Å (for CH_2_) and refined using a riding model by applying the constraint *U*_iso_(H) = 1.2*U*_eq_(C).

## Supplementary Material

Crystal structure: contains datablock(s) I. DOI: 10.1107/S2056989025004451/ee2014sup1.cif

Structure factors: contains datablock(s) I. DOI: 10.1107/S2056989025004451/ee2014Isup2.hkl

Supporting information file. DOI: 10.1107/S2056989025004451/ee2014Isup3.cml

CCDC reference: 2452369

Additional supporting information:  crystallographic information; 3D view; checkCIF report

## Figures and Tables

**Table 1:** Hydrogen-bond geometry (Å, °)

*D*—H⋯*A*	*D*—H	H⋯*A*	*D*⋯*A*	*D*—H⋯*A*
O1—H1⋯N1^i^	0.853 (17)	1.997 (16)	2.8440 (18)	171.6 (14)

Symmetry code: (i) 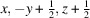.

**Table 2:** Selected interatomic distances (Å)

O1⋯N1	2.9300 (19)	H1⋯C2^i^	2.841 (16)
O1⋯N1^i^	2.8440 (18)	C3⋯H16	2.65
N1⋯H5	2.62	C16⋯H3	2.63
H1⋯N1^i^	1.997 (16)	H1⋯H5^i^	2.33
C3⋯C19^ii^	3.386 (2)	H2*A*⋯H3	2.07
C17⋯C17^ii^	3.396 (2)	H3⋯H16	2.06

Symmetry codes: (i) 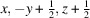; (ii) 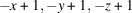.

**Table 3:** Experimental details

Crystal data	
Chemical formula	C_19_H_15_NO
*M*_r_	273.32
Crystal system, space group	Monoclinic, *P*2_1_/*c*
Temperature (K)	100
*a*, *b*, *c* (Å)	7.447 (2), 20.916 (8), 8.446 (3)
β (°)	93.347 (9)
*V* (Å^3^)	1313.3 (8)
*Z*	4
Radiation type	Mo *K*α
μ (mm^−1^)	0.09
Crystal size (mm)	0.37 × 0.35 × 0.28

Data collection	
Diffractometer	Bruker D8 Quest
Absorption correction	Multi-scan (*SADABS*; Krause *et al.*, 2015 ▸)
No. of measured, independent and observed [*I* > 2σ(*I*)] reflections	14470, 2362, 1980
*R*_int_	0.044
(sin θ/λ)_max_ (Å^−1^)	0.601

Refinement	
*R*[*F*^2^ > 2σ(*F*^2^)], *wR*(*F*^2^), *S*	0.038, 0.106, 1.04
No. of reflections	2362
No. of parameters	193
H-atom treatment	H atoms treated by a mixture of independent and constrained refinement
Δρ_max_, Δρ_min_ (e Å^−3^)	0.18, −0.25

Computer programs: *APEX3* and *SAINT* (Bruker, 2021 ▸), *SHELXT2014/5* (Sheldrick, 2015*a* ▸), *SHELXL2018/3* (Sheldrick, 2015*b* ▸) and *SHELXTL* (Sheldrick, 2008 ▸).

## supplementary crystallographic information

### (E)-2-[(Pyren-1-ylmethylidene)amino]ethanol Crystal data

**Table:**

C_19_H_15_NO	*F*(000) = 576
*M**_r_* = 273.32	*D*_x_ = 1.382 Mg m^−^^3^
Monoclinic, *P*2_1_/*c*	Mo *K*α radiation, λ = 0.71073 Å
*a* = 7.447 (2) Å	Cell parameters from 7593 reflections
*b* = 20.916 (8) Å	θ = 2.6–25.4°
*c* = 8.446 (3) Å	µ = 0.09 mm^−^^1^
β = 93.347 (9)°	*T* = 100 K
*V* = 1313.3 (8) Å^3^	Block, colourless
*Z* = 4	0.37 × 0.35 × 0.28 mm

### (E)-2-[(Pyren-1-ylmethylidene)amino]ethanol Data collection

**Table:**

Bruker D8 Quest diffractometer	1980 reflections with *I* > 2σ(*I*)
φ and ω scans	*R*_int_ = 0.044
Absorption correction: multi-scan (SADABS; Krause *et al.*, 2015)	θ_max_ = 25.3°, θ_min_ = 2.7°
	*h* = −8→8
14470 measured reflections	*k* = −25→25
2362 independent reflections	*l* = −10→10

### (E)-2-[(Pyren-1-ylmethylidene)amino]ethanol Refinement

**Table:**

Refinement on *F*^2^	0 restraints
Least-squares matrix: full	Hydrogen site location: mixed
*R*[*F*^2^ > 2σ(*F*^2^)] = 0.038	H atoms treated by a mixture of independent and constrained refinement
*wR*(*F*^2^) = 0.106	*w* = 1/[σ^2^(*F*_o_^2^) + (0.0576*P*)^2^ + 0.4126*P*] where *P* = (*F*_o_^2^ + 2*F*_c_^2^)/3
*S* = 1.04	(Δ/σ)_max_ = 0.019
2362 reflections	Δρ_max_ = 0.18 e Å^−^^3^
193 parameters	Δρ_min_ = −0.25 e Å^−^^3^

### (E)-2-[(Pyren-1-ylmethylidene)amino]ethanol Special details

**Table:**

Geometry. All esds (except the esd in the dihedral angle between two l.s. planes) are estimated using the full covariance matrix. The cell esds are taken into account individually in the estimation of esds in distances, angles and torsion angles; correlations between esds in cell parameters are only used when they are defined by crystal symmetry. An approximate (isotropic) treatment of cell esds is used for estimating esds involving l.s. planes.

### (E)-2-[(Pyren-1-ylmethylidene)amino]ethanol Fractional atomic coordinates and isotropic or equivalent isotropic displacement parameters (Å^2^)

**Table:**

	*x*	*y*	*z*	*U*_iso_*/*U*_eq_	
O1	0.20414 (13)	0.21597 (5)	0.52257 (12)	0.0216 (3)	
H1	0.248 (2)	0.2057 (8)	0.616 (2)	0.032*	
N1	0.37839 (14)	0.30976 (5)	0.32442 (13)	0.0152 (3)	
C1	0.32460 (18)	0.19901 (6)	0.40609 (16)	0.0171 (3)	
H1A	0.256590	0.191054	0.306579	0.020*	
H1B	0.385335	0.159619	0.437830	0.020*	
C2	0.46484 (18)	0.25029 (6)	0.38038 (16)	0.0164 (3)	
H2A	0.534355	0.258221	0.479141	0.020*	
H2B	0.546544	0.235486	0.302886	0.020*	
C3	0.38451 (17)	0.35642 (6)	0.42167 (15)	0.0146 (3)	
H3	0.444841	0.350163	0.519987	0.017*	
C4	0.30263 (16)	0.41963 (6)	0.38920 (15)	0.0130 (3)	
C5	0.23407 (17)	0.43504 (6)	0.23570 (15)	0.0145 (3)	
H5	0.237547	0.404567	0.155754	0.017*	
C6	0.16132 (16)	0.49467 (6)	0.20070 (15)	0.0143 (3)	
H6	0.117589	0.503528	0.097642	0.017*	
C7	0.15235 (16)	0.54192 (6)	0.31769 (15)	0.0128 (3)	
C8	0.07710 (17)	0.60437 (6)	0.28453 (15)	0.0154 (3)	
H8	0.035518	0.614331	0.181508	0.019*	
C9	0.06565 (17)	0.64883 (6)	0.40009 (16)	0.0167 (3)	
H9	0.016214	0.688652	0.375061	0.020*	
C10	0.12884 (16)	0.63543 (6)	0.56126 (15)	0.0140 (3)	
C11	0.11339 (17)	0.68037 (6)	0.68337 (17)	0.0174 (3)	
H11	0.060084	0.719801	0.660935	0.021*	
C12	0.17704 (17)	0.66639 (6)	0.83720 (16)	0.0179 (3)	
H12	0.166897	0.696676	0.916797	0.021*	
C13	0.25590 (17)	0.60745 (6)	0.87342 (15)	0.0167 (3)	
H13	0.299764	0.599066	0.976686	0.020*	
C14	0.27000 (16)	0.56069 (6)	0.75641 (15)	0.0138 (3)	
C15	0.34525 (17)	0.49843 (6)	0.78892 (15)	0.0149 (3)	
H15	0.386896	0.488566	0.891982	0.018*	
C16	0.35730 (17)	0.45384 (6)	0.67445 (15)	0.0146 (3)	
H16	0.407480	0.414277	0.701019	0.018*	
C17	0.29455 (16)	0.46569 (6)	0.51163 (15)	0.0125 (3)	
C18	0.21810 (16)	0.52728 (6)	0.47568 (15)	0.0122 (3)	
C19	0.20622 (16)	0.57444 (6)	0.59733 (15)	0.0126 (3)	

### (E)-2-[(Pyren-1-ylmethylidene)amino]ethanol Atomic displacement parameters (Å^2^)

**Table:**

	*U*^11^	*U*^22^	*U*^33^	*U*^12^	*U*^13^	*U*^23^
O1	0.0190 (5)	0.0258 (6)	0.0203 (5)	0.0037 (4)	0.0046 (4)	0.0078 (4)
N1	0.0156 (6)	0.0122 (6)	0.0180 (6)	−0.0018 (4)	0.0029 (4)	0.0010 (4)
C1	0.0208 (7)	0.0129 (7)	0.0173 (7)	0.0002 (5)	−0.0013 (5)	0.0012 (5)
C2	0.0168 (7)	0.0151 (7)	0.0175 (7)	0.0025 (5)	0.0021 (5)	−0.0003 (5)
C3	0.0127 (6)	0.0167 (7)	0.0145 (6)	−0.0029 (5)	0.0020 (5)	0.0011 (5)
C4	0.0098 (6)	0.0138 (7)	0.0156 (7)	−0.0029 (5)	0.0022 (5)	0.0017 (5)
C5	0.0141 (7)	0.0148 (7)	0.0150 (7)	−0.0027 (5)	0.0028 (5)	−0.0022 (5)
C6	0.0119 (6)	0.0191 (7)	0.0118 (6)	−0.0016 (5)	−0.0009 (5)	0.0029 (5)
C7	0.0086 (6)	0.0162 (6)	0.0139 (6)	−0.0028 (5)	0.0020 (5)	0.0024 (5)
C8	0.0129 (6)	0.0187 (7)	0.0146 (6)	−0.0001 (5)	0.0001 (5)	0.0049 (5)
C9	0.0131 (6)	0.0135 (6)	0.0235 (7)	0.0007 (5)	0.0020 (5)	0.0054 (5)
C10	0.0099 (6)	0.0143 (7)	0.0181 (7)	−0.0030 (5)	0.0030 (5)	0.0001 (5)
C11	0.0129 (6)	0.0146 (7)	0.0248 (8)	−0.0007 (5)	0.0031 (5)	−0.0008 (5)
C12	0.0156 (7)	0.0174 (7)	0.0209 (7)	−0.0036 (5)	0.0039 (5)	−0.0063 (6)
C13	0.0142 (7)	0.0227 (7)	0.0134 (7)	−0.0049 (5)	0.0015 (5)	−0.0014 (5)
C14	0.0093 (6)	0.0160 (7)	0.0162 (7)	−0.0039 (5)	0.0023 (5)	0.0004 (5)
C15	0.0127 (6)	0.0198 (7)	0.0119 (6)	−0.0026 (5)	−0.0006 (5)	0.0022 (5)
C16	0.0135 (6)	0.0129 (6)	0.0173 (7)	−0.0003 (5)	0.0001 (5)	0.0043 (5)
C17	0.0093 (6)	0.0138 (7)	0.0144 (6)	−0.0036 (5)	0.0021 (5)	0.0018 (5)
C18	0.0079 (6)	0.0144 (7)	0.0146 (7)	−0.0034 (5)	0.0023 (5)	0.0014 (5)
C19	0.0082 (6)	0.0155 (7)	0.0143 (7)	−0.0036 (5)	0.0031 (5)	0.0002 (5)

### (E)-2-[(Pyren-1-ylmethylidene)amino]ethanol Geometric parameters (Å, º)

**Table:**

O1—C1	1.4148 (17)	C8—H8	0.9300
O1—H1	0.862 (19)	C9—C10	1.4415 (19)
N1—C3	1.2745 (17)	C9—H9	0.9300
N1—C2	1.4662 (17)	C10—C11	1.4051 (19)
C1—C2	1.5215 (19)	C10—C19	1.4254 (19)
C1—H1A	0.9700	C11—C12	1.388 (2)
C1—H1B	0.9700	C11—H11	0.9300
C2—H2A	0.9700	C12—C13	1.3918 (19)
C2—H2B	0.9700	C12—H12	0.9300
C3—C4	1.4750 (18)	C13—C14	1.3988 (19)
C3—H3	0.9300	C13—H13	0.9300
C4—C5	1.4029 (19)	C14—C19	1.4279 (19)
C4—C17	1.4171 (19)	C14—C15	1.4378 (19)
C5—C6	1.3847 (19)	C15—C16	1.3500 (19)
C5—H5	0.9300	C15—H15	0.9300
C6—C7	1.4019 (19)	C16—C17	1.4476 (18)
C6—H6	0.9300	C16—H16	0.9300
C7—C18	1.4275 (18)	C17—C18	1.4337 (19)
C7—C8	1.4424 (18)	C18—C19	1.4307 (19)
C8—C9	1.3543 (19)		
			
O1···N1	2.9300 (19)	H1···C2^i^	2.841 (16)
O1···N1^i^	2.8440 (18)	C3···H16	2.65
N1···H5	2.62	C16···H3	2.63
H1···N1^i^	1.997 (16)	H1···H5^i^	2.33
C3···C19^ii^	3.386 (2)	H2A···H3	2.07
C17···C17^ii^	3.396 (2)	H3···H16	2.06
			
C1—O1—H1	110.7 (12)	C8—C9—H9	119.5
C3—N1—C2	116.32 (11)	C10—C9—H9	119.5
O1—C1—C2	113.01 (11)	C11—C10—C19	119.43 (12)
O1—C1—H1A	109.0	C11—C10—C9	121.79 (12)
C2—C1—H1A	109.0	C19—C10—C9	118.78 (12)
O1—C1—H1B	109.0	C12—C11—C10	120.53 (13)
C2—C1—H1B	109.0	C12—C11—H11	119.7
H1A—C1—H1B	107.8	C10—C11—H11	119.7
N1—C2—C1	110.61 (11)	C11—C12—C13	120.60 (12)
N1—C2—H2A	109.5	C11—C12—H12	119.7
C1—C2—H2A	109.5	C13—C12—H12	119.7
N1—C2—H2B	109.5	C12—C13—C14	120.81 (12)
C1—C2—H2B	109.5	C12—C13—H13	119.6
H2A—C2—H2B	108.1	C14—C13—H13	119.6
N1—C3—C4	124.59 (12)	C13—C14—C19	119.30 (12)
N1—C3—H3	117.7	C13—C14—C15	122.89 (12)
C4—C3—H3	117.7	C19—C14—C15	117.82 (12)
C5—C4—C17	119.30 (12)	C16—C15—C14	122.03 (12)
C5—C4—C3	120.13 (12)	C16—C15—H15	119.0
C17—C4—C3	120.56 (12)	C14—C15—H15	119.0
C6—C5—C4	121.40 (12)	C15—C16—C17	122.05 (12)
C6—C5—H5	119.3	C15—C16—H16	119.0
C4—C5—H5	119.3	C17—C16—H16	119.0
C5—C6—C7	121.24 (12)	C4—C17—C18	119.31 (12)
C5—C6—H6	119.4	C4—C17—C16	123.47 (12)
C7—C6—H6	119.4	C18—C17—C16	117.21 (12)
C6—C7—C18	118.67 (12)	C7—C18—C19	119.41 (12)
C6—C7—C8	122.39 (12)	C7—C18—C17	120.07 (12)
C18—C7—C8	118.94 (12)	C19—C18—C17	120.52 (12)
C9—C8—C7	121.47 (12)	C10—C19—C14	119.31 (12)
C9—C8—H8	119.3	C10—C19—C18	120.32 (12)
C7—C8—H8	119.3	C14—C19—C18	120.38 (12)
C8—C9—C10	121.07 (12)		
			
C3—N1—C2—C1	110.27 (13)	C3—C4—C17—C18	178.50 (10)
O1—C1—C2—N1	−61.54 (14)	C5—C4—C17—C16	178.35 (11)
C2—N1—C3—C4	−178.71 (11)	C3—C4—C17—C16	−2.55 (19)
N1—C3—C4—C5	−9.67 (19)	C15—C16—C17—C4	−178.97 (12)
N1—C3—C4—C17	171.23 (12)	C15—C16—C17—C18	0.00 (18)
C17—C4—C5—C6	1.06 (19)	C6—C7—C18—C19	−178.58 (11)
C3—C4—C5—C6	−178.05 (11)	C8—C7—C18—C19	0.64 (17)
C4—C5—C6—C7	−0.38 (19)	C6—C7—C18—C17	1.17 (17)
C5—C6—C7—C18	−0.74 (18)	C8—C7—C18—C17	−179.62 (11)
C5—C6—C7—C8	−179.92 (11)	C4—C17—C18—C7	−0.50 (18)
C6—C7—C8—C9	178.35 (12)	C16—C17—C18—C7	−179.52 (10)
C18—C7—C8—C9	−0.83 (18)	C4—C17—C18—C19	179.24 (10)
C7—C8—C9—C10	0.13 (19)	C16—C17—C18—C19	0.23 (18)
C8—C9—C10—C11	−178.20 (12)	C11—C10—C19—C14	−1.30 (18)
C8—C9—C10—C19	0.74 (18)	C9—C10—C19—C14	179.74 (11)
C19—C10—C11—C12	1.71 (19)	C11—C10—C19—C18	178.05 (11)
C9—C10—C11—C12	−179.36 (12)	C9—C10—C19—C18	−0.91 (18)
C10—C11—C12—C13	−0.53 (19)	C13—C14—C19—C10	−0.27 (18)
C11—C12—C13—C14	−1.1 (2)	C15—C14—C19—C10	179.26 (11)
C12—C13—C14—C19	1.47 (19)	C13—C14—C19—C18	−179.61 (11)
C12—C13—C14—C15	−178.03 (11)	C15—C14—C19—C18	−0.09 (17)
C13—C14—C15—C16	179.82 (12)	C7—C18—C19—C10	0.23 (18)
C19—C14—C15—C16	0.32 (18)	C17—C18—C19—C10	−179.52 (11)
C14—C15—C16—C17	−0.28 (19)	C7—C18—C19—C14	179.57 (11)
C5—C4—C17—C18	−0.61 (18)	C17—C18—C19—C14	−0.18 (18)

Symmetry codes: (i) *x*, −*y*+1/2, *z*+1/2; (ii) −*x*+1, −*y*+1, −*z*+1.

### (E)-2-[(Pyren-1-ylmethylidene)amino]ethanol Hydrogen-bond geometry (Å, º)

**Table:**

*D*—H···*A*	*D*—H	H···*A*	*D*···*A*	*D*—H···*A*
O1—H1···N1^i^	0.853 (17)	1.997 (16)	2.8440 (18)	171.6 (14)

Symmetry code: (i) *x*, −*y*+1/2, *z*+1/2.
